# Novel Alkaloids from Marine Actinobacteria: Discovery and Characterization

**DOI:** 10.3390/md20010006

**Published:** 2021-12-22

**Authors:** Anne-Sofie De Rop, Jeltien Rombaut, Thomas Willems, Marilyn De Graeve, Lynn Vanhaecke, Paco Hulpiau, Sofie L. De Maeseneire, Maarten L. De Mol, Wim K. Soetaert

**Affiliations:** 1 Centre for Industrial Biotechnology and Biocatalysis (InBio.be), Department of Biotechnology, Faculty of Bioscience Engineering, Ghent University, Coupure Links 653, 9000 Ghent, Belgium; AnneSofie.DeRop@UGent.be (A.-S.D.R.); Jeltien.Rombaut@UGent.be (J.R.); Thomas.Willems@UGent.be (T.W.); Maarten.DeMol@UGent.be (M.L.D.M.); Wim.Soetaert@UGent.be (W.K.S.); 2 Laboratory of Chemical Analysis (LCA), Department of Translational Physiology, Infectiology and Public Health, Faculty of Veterinary Medicine, Ghent University, Salisburylaan 133, 9820 Merelbeke, Belgium; Marilyn.DeGraeve@UGent.be (M.D.G.); Lynn.Vanhaecke@UGent.be (L.V.); 3 BioInformatics Knowledge Center (BiKC), Campus Station Brugge, Howest University of Applied Sciences, Rijselstraat 5, 8200 Bruges, Belgium; Paco.Hulpiau@howest.be

**Keywords:** marine Actinobacteria, alkaloids, chemical structure elucidation, genome mining, synthetic biology, biosynthesis

## Abstract

The marine environment is an excellent resource for natural products with therapeutic potential. Its microbial inhabitants, often associated with other marine organisms, are specialized in the synthesis of bioactive secondary metabolites. Similar to their terrestrial counterparts, marine Actinobacteria are a prevalent source of these natural products. Here, we discuss 77 newly discovered alkaloids produced by such marine Actinobacteria between 2017 and mid-2021, as well as the strategies employed in their elucidation. While 12 different classes of alkaloids were unraveled, indoles, diketopiperazines, glutarimides, indolizidines, and pyrroles were most dominant. Discoveries were mainly based on experimental approaches where microbial extracts were analyzed in relation to novel compounds. Although such experimental procedures have proven useful in the past, the methodologies need adaptations to limit the chance of compound rediscovery. On the other hand, genome mining provides a different angle for natural product discovery. While the technology is still relatively young compared to experimental screening, significant improvement has been made in recent years. Together with synthetic biology tools, both genome mining and extract screening provide excellent opportunities for continued drug discovery from marine Actinobacteria.

## 1. Introduction

Natural products, small molecules isolated from biological sources, have long been recognized for their huge potential in human medicine and are still gaining traction in recent years. The advent of novel technologies, such as more precise analytical methods or improved genome mining approaches, has propelled natural product discovery ahead of (combinatorial) chemistry in the field of drug discovery [1]. In general, natural products often possess less nitrogen and halogen atoms compared with synthetically designed compounds [2]. The marine environment, however, bears sundry conditions in salinity and nutrients. Combined with varied conditions of temperature, pressure, acidity, and light, the marine environment is a true cauldron for novel natural product discovery [3], including compounds with naturally rare atoms. Despite such versatile environmental conditions yielding a plethora of unique secondary metabolites and the up to four times higher success rate in drug discovery, marine natural products are still underrepresented [4]. To date, as little as around 35,000 marine natural products have been discovered. With a yearly incremental increase of 1500 novel compounds, this environment is finally receiving the attention it deserves [5].

Marine alkaloids are a therapeutically interesting class of compounds, with proven anti-tumor, inflammation-mediating, and pain-relieving properties, as thoroughly reviewed by Lu et al. [6]. In addition to clinical trials, an increasing number of in vivo studies with marine alkaloids are being reported, e.g., for the treatment of cardiovascular diseases, mental disorders, osteoporosis, and (microbial) infections [7]. Marine Actinobacteria, a prevalent source of such alkaloids, are often associated with other marine organisms, such as animals, plants, algae, cyanobacteria, and lichen as their armamentarium of secondary metabolites bolsters the associated host’s defenses against predators and/or microbial infections [8]. Up to 37% of their reported secondary metabolites belong to the alkaloid class [8].

While numerous reviews about marine Actinobacteria and their secondary metabolites address the compounds in general (e.g., halogenated molecules [9]) or revise their biological functionalities (e.g., biofilm inhibition [10]), an overview of recently discovered alkaloids produced by marine Actinobacteria is missing. We have, therefore, dived into literature from 2017 till mid-2021 to provide a summary of such literature along with the employed experimental and genome mining approaches. Finally, we discuss the power of synthetic biology to bridge the gap between compound discovery and production.

## 2. Novel Alkaloids from Marine Actinobacteria

This section sums up which naturally produced marine alkaloids were reported from 2017 till mid-2021, together with the place where they were discovered, the natural production host, the activities for which they were evaluated, and hints for their biosynthesis, when found. Literature on microbial alkaloid biochemistry is rather limited [11,12,13,14] compared with alkaloids from plant sources. Unless otherwise mentioned, the compounds were discovered after analysis of microbial extracts. A summary of the available information on the newly discovered alkaloids is given in Supplementary Table S1.

### 2.1. Piperidines

**Figure 1:**
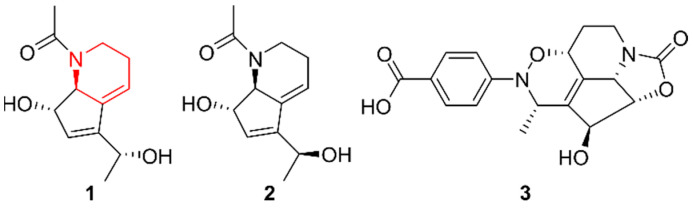
The piperidine alkaloids strepchazolin A and B (**1**–**2**) and chartrenoline (**3**). The structural alkaloid group is represented in red.

Since 2017, three new piperidine alkaloids have been identified from marine Actinobacteria (Figure 1). Strepchazolin A and B (**1**–**2**) were discovered by Yang et al. (2017) in *Streptomyces chartreusis* NA02069, collected at the coast of Hainan Island (China) [15]. While compound **1** possesses weak antibacterial activity against *Bacillus subtilis* and inhibits acetylcholinesterase (AChE), compound **2** is almost inactive. As the difference in chemical structure between both compounds is a single hydroxyl group, hydroxylation of a piperidine backbone might prove beneficial for therapeutic application. In 2019, the same compounds **1** and **2** were isolated from *Streptomyces chartreusis* ICBG377, discovered in a fungal garden on the *Acromyrmex subterraneus brunneus* ant [16]. Though originating from totally different natural habitats, both strains were able to produce the same piperidine alkaloids under laboratory conditions. Continued investigation of the *Streptomyces chartreusis* NA02069 isolate revealed another novel piperidine alkaloid, chartrenoline (**3**), in 2019 [17]. This compound was screened for antibacterial activity against *Staphylococcus aureus*, *B. subtilis*, *Escherichia coli*, and *Pseudomonas aeruginosa*, yet was inactive against all four. While more extensive application testing, such as the existence of antifungal or cytotoxic properties, is lacking, a hypothetical biosynthesis pathway was predicted. Due to the structural similarity of compound **3** to streptazolin, a Diels–Alder cycloaddition of 4-nitrosobenzoic acid and streptazolin was proposed, leading to the formation of **3.** Bacterial type I polyketide synthases (PKSs) were proposed to be involved during biosynthesis of streptazolin, with the C-terminal thioester reductase domain responsible for releasing the polyketide strain as an aldehyde [18]. Afterwards, reductive amination and cyclization led to the formation of streptazolin, which was hypothesized to play a role during the biosynthesis of **3**.

### 2.2. Glutarimides

**Figure 2:**
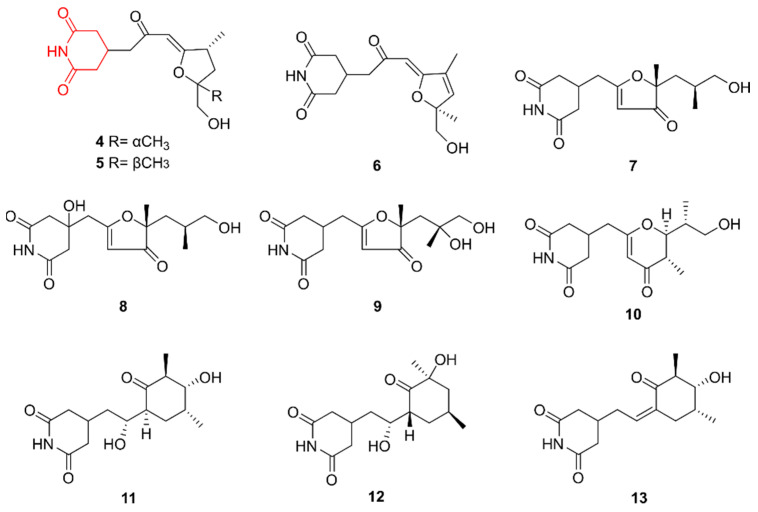
The glutarimide alkaloids streptoglutarimides A–J (**4**–**13**). The structural alkaloid group is represented in red.

Glutarimide alkaloids share the same core structure as piperidines but have two oxide groups bound to the ring structure. They are characterized by diverse side chains at the C-4 position of the glutarimide ring [19]. Zhang et al. (2019) isolated streptoglutarimides A–J (**4**–**13**) from *Streptomyces* sp. ZZ741, a strain isolated in 2016 from marine mud in Zhoushan, China [20]. Chemical investigation led to the discovery of **4**–**13** after observing the inhibitory effects of the extract of the strain on glioma cells and methicillin-resistant *Staphylococcus aureus* (MRSA). All compounds exhibited antibacterial activities against MRSA and *E. coli* and antifungal activity against *Candida albicans*, while **11** showed inhibitory effects on the proliferation of human glioma cells U87MG and U251. The structure of these compounds is presented in Figure 2.

### 2.3. Indolizidines

Compared to piperidines, indolizidine alkaloids have been isolated more frequently from marine Actinobacteria in recent years. Interestingly, indolizinium alkaloids, which are indolizidines with a positive charge on the heterocyclic nitrogen, were well-represented, despite being rare actinomycete secondary metabolites first discovered in 2016 [21].

The indolizinium alkaloid streptopertusacin A (**14**) was isolated from *Streptomyces* sp. HZP-2216E by Zhang et al. (2017) from fresh sea lettuce *Ulva pertusaas* growing on rocks located in South China [22]. In addition to being an indolizinium alkaloid, compound **14** is also a zwitterion. It was tested for inhibitory effects on the proliferation of glioma U251 and C6 cells as well as against MRSA but showed only activity against the latter, with a minimal inhibitory concentration (MIC) of 40 µg/mL.

**Figure 3:**
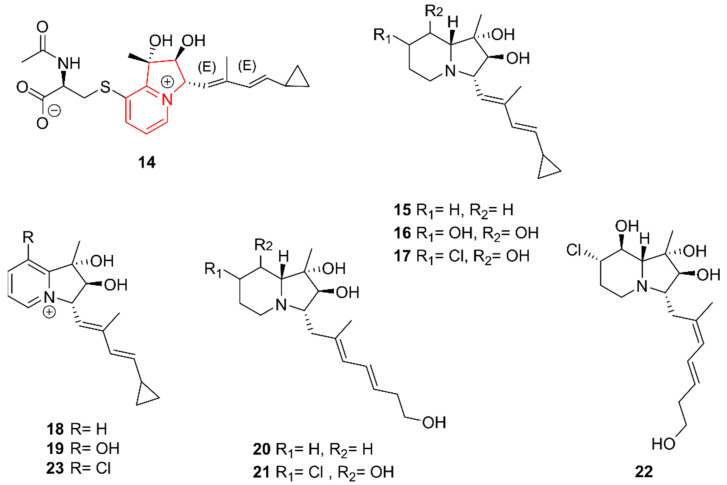
The indolizidine alkaloids streptopertusacin A (**14**) and cyclizidines B–J (**15**–**23**). The structural alkaloid group is represented in red.

Cyclizidines B–I (**15**–**22**) were derived by Jiang et al. (2018) from *Streptomyces* sp. HNA39 [23]. This strain was isolated from marine sediments on Hainan Island in China. With the exception of **18** and **19**, which are indolizinium alkaloids, all compounds were regular indolizidines. Compounds **16**, **19**, **21**, and **22** moderately inhibited the ROCK2 protein kinase, which is proven to be beneficial when combatting neurodegenerative disorders such as Alzheimer’s disease [24]. Only **16** was significantly cytotoxic against PC3 and HCT116 human cancer cell lines. Although the compounds’ cytotoxicity and inhibitory effects were studied, their antimicrobial activity was not. In 2020, the family of indolizinium alkaloids was further extended with cyclizidine J (**23**), isolated from the same *Streptomyces* sp. HNA39 strain [25]. Showing the opposite effect to cyclizidines **15**–**22**, **23** harbored a chlorine atom, making it unique. No therapeutic activity was observed for **23**. The structures of the newly discovered indolizidine alkaloids are presented in Figure 3.

### 2.4. Pyrroles

**Figure 4:**
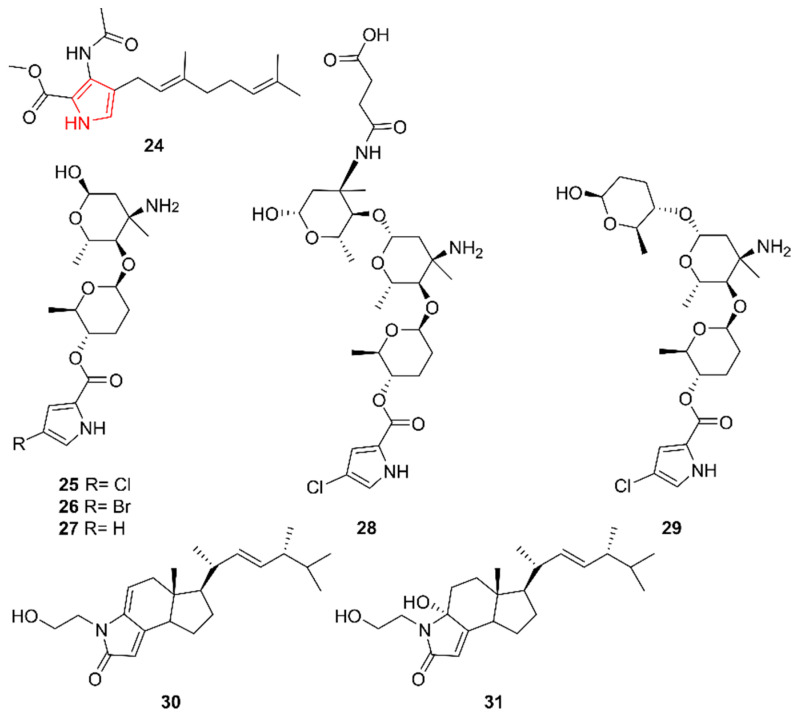
The pyrrole alkaloids geranylpyrrol A (**24**), phallusialides A–E (**25**–**29**), and anandins A and B (**30**–**31**). The structural alkaloid group is represented in red.

Pyrroles are prevalently isolated from marine organisms, reflected by a recent review focusing on pyrroles from marine sources covering the decade from 2010 to 2020 [26]. While most marine pyrroles originate from sponges, several actinomycetes were also found to be hosts to novel pyrroles (Figure 4).

Geranylpyrrol A (**24**) was isolated by Han et al. (2017) from a mutant strain of *Streptomyces* sp. CHQ-64 [27]. The wild type actinomycete was isolated from the reed rhizosphere soil of a mangrove in Guangdong Province, China. To increase the likelihood of unravelling novel molecules, a regulatory gene for the biosynthesis of the abundantly present macrolide reedsmycin was knocked out, yielding the mutant strain. Though tested against HeLa, A549, H1975, MCF-7/ADM, HTC-116, NB4, K562, and HL-60 cells, no cytotoxic activity was perceived for compound **24**. It is believed to have originated from the condensation of an α-pyrrole and the terpene geranyl. Although numerous aromatic ring structures combined with terpenoid substructures exist, pyrrole-linked terpene structures are rather rare and have yielded little biochemical information on these compounds [28].

In 2019, Zhang et al. discovered phallusialides A–E (**25**–**29**), produced by *Micromonospora* sp. WMMA-2495, isolated from the tunicate *Phallusia nigra* in the Florida Keys [29]. From 72 extracts of *Micromonospora* sp. Strain, WMMA-2495 an outlier was detected and further analyzed using molecular networking. Antimicrobial activity tests revealed that only **25** and **26** were active against MRSA and *E. coli* with a MIC of 32 µg/mL and 64 µg/mL, respectively.

Occasionally, steroidal structures with a nitrogen atom incorporated in their skeleton, resulting in a pyrrole alkaloid, are discovered. Recently, two such compounds were found in marine actinomycetes. Anandins A and B (**30**–**31**) were discovered by Zhang et al. in 2017 in an extract from *Streptomyces anandii* H41-59 isolated from a sea sediment sample in a mangrove zone in the South China Sea [30]. The alkaloids were screened for activity against *C. albicans*, *E. coli*, *S. aureus*, *Bacillus* sp., and *Dickeya zeae*, but no antimicrobial activity was observed. In contrast, cytotoxic activity screening against the human breast adenocarcinoma cell line MCF-7, human glioblastoma cell line SF-268, and human lung cancer cell line NCI-H460 yielded positive results for both compounds, wherein **30** was the most effective.

### 2.5. Pyrrolidines

**Figure 5:**
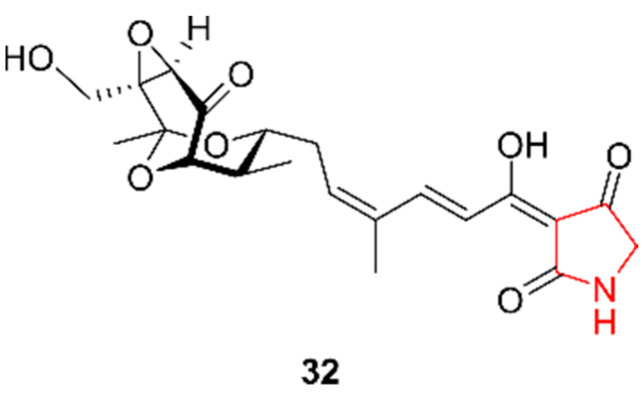
The pyrrolidine alkaloid isotirandamycin B (**32**). The structural alkaloid group is represented in red.

Pyrrolidines are structurally similar to pyrroles, yet only one pyrrolidine was discovered in marine actinomycetes. Since 2017, this class of alkaloids was most prevalently discovered in marine fungi [11]. Cong et al. (2018) isolated isotirandamycin B (**32**) from *Streptomyces* sp. SCSIO 41399, a strain retrieved in 2016 from coral *Porites* sp. located in Wenchang, China [31]. The compound (Figure 5) was screened for antibacterial activity and was found to be active against *Streptococcus agalactiae*. In addition, antifungal activities against *Colletotrichum gloeosporioides*, *Colletotrichum asianum*, *Colletotrichum acutatum*, *Fusarium oxysporum*, and *Pleomorphomonas oryza* was evaluated, but no activity was detected. Moreover, **32** exhibited no cytotoxic effects on K562 and BEL-7402 cells.

### 2.6. Pyridines

**Figure 6:**
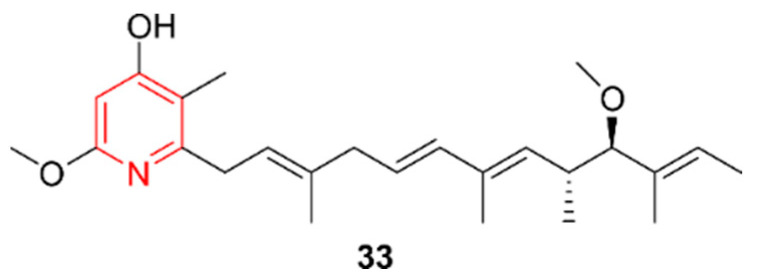
The pyridine alkaloid piericidin F (**33**). The structural alkaloid group is represented in red.

Pyridines can target a wide range of cellular components, including topoisomerases and PIM-1 kinases (both enzymes involved during cancer development), making them interesting targets for inhibition during treatment [32]. In parallel with the discovery of geranylpyrrol A (**24**), Han et al. (2017) discovered piericidin F (**33**, Figure 6) produced by the same mutant strain *Streptomyces* sp. CHQ-64 Δ*rdm* [27]. This alkaloid was subjected to the same screening tests as geranylpyrrol A and showed cytotoxicity against the HeLa, NB4, A549, and H1975 cell lines, with half maximal inhibitory concentration (IC_50_) values of 1.25 ng/mL, 15.38 ng/mL, 232.74 ng/mL, and 203.64 ng/µL, respectively.

### 2.7. Imidazoles

Since 2017, three new imidazoles have been discovered in marine actinomycetes. Chen et al. (2019) identified 2-ethylhexyl 1H-imidazole-4-carboxylate (**34**) from *Verrucosispora* sp. FIM06-0036, a strain isolated from a marine sponge in the East China Sea [33]. Antimicrobial activities were observed against *Helicobacter pylori*, *Klebsiella pneumonia*, *S*. *aureus*, and *Enterococcus faecium*, with MICs of 8 µg/mL 64 µg/mL, 16 µg/mL, and 256 µg/mL, respectively.

Nocarimidazoles C and D (**35**, **36**) were isolated from *Kocuria* sp. T35-5 retrieved from the coral *Mycedium* sp. collected at the Karimunjawa National Park in Indonesia [34]. Both compounds are moderately active against the Gram-positive bacteria *Kocuria rhizophila* and *S. aureus*, with a MIC ranging from 6.25 to 12.5 µg/mL for compound **35** and from 12.5 to 25 µg/mL for compound **36**. Activity against the Gram-negative bacteria *E. coli* and *Rhizobium radiobacter* was absent. Additionally, **35** showed weak cytotoxic activity against P388 murine leukemia cells with an IC_50_ of 9.02 µg/mL. Finally, antifungal activity against *Glomerella cingulate* was detected, with a MIC of 12.5 µg/mL for both compounds, and activity against *Trichophyton rubrum* was observed for both, with a MIC of 6.25 µg/mL.

**Figure 7:**
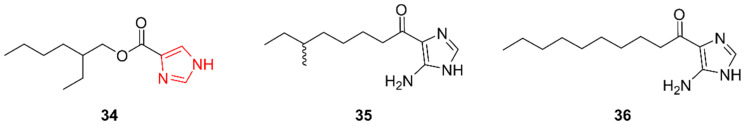
The imidazole alkaloids 2-ethylhexyl 1H-imidazole-4-carboxylate (**34**) and nocarimidazoles C and D (**35**, **36**). The structural alkaloid group is represented in red.

The structures of these compounds are presented in Figure 7.

### 2.8. Pyrimidines

**Figure 8:**
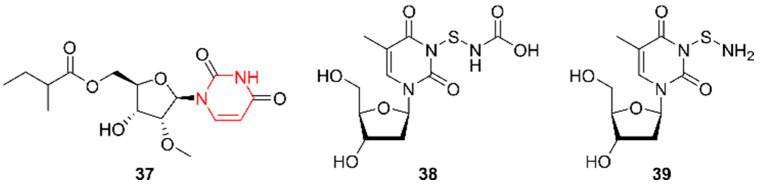
The pyrimidine alkaloids 11457A (**37**), thymidine-3-mercaptocarbamic acid (**38**), and thymidine-3-thioamine (**39**)**.** The structural alkaloid group is represented in red.

Fang et al., in a report published in 2019, identified the uridine derivative 11457A (**37**, Figure 8) in the extracts from the actinomycete *Pseudonocardia* sp. SCSIO 11457, isolated from the coral *Galaxea fascicularis* from the Luhuitou fringing reef in the South China Sea at a depth of 3–5 m in 2011 [35]. Nucleoside analogues are known to have great potential in cancer treatment, as they can act as imposters during DNA replication and in DNA repair systems [36]. However, often only their triphosphate analogues are active. Compound **37** was screened for cytotoxic activity against cell lines SF-268, MCF-7, NCI-H460, A549, and L0-2 and for antibacterial activities against the coral pathogen *Vibrio alginolyticus*, *S. aureus*, MRSA, and *Acinetobacter baumannii*. It was inactive at all levels. A possible explanation resides in the absence of the **37**-triphosphate analogue in vivo due to the presence of an acyl group.

In 2019, Shaala et al. discovered two additional nucleoside analogues, namely thymidine-3-mercaptocarbamic acid (**38**) and thymidine-3-thioamine (**39**), from *Streptomyces* species Call-36 (Figure 8) [37]. This actinomycete strain was isolated from the Red Sea sponge *Callyspongia*. Both compounds were evaluated for their cytotoxic activities against HCT-116 (colorectal carcinoma) and MCF-7 (breast cancer) and exhibited only weak activity against human breast cells. The antimicrobial properties of these compounds were not evaluated.

### 2.9. Indoles

**Figure 9:**
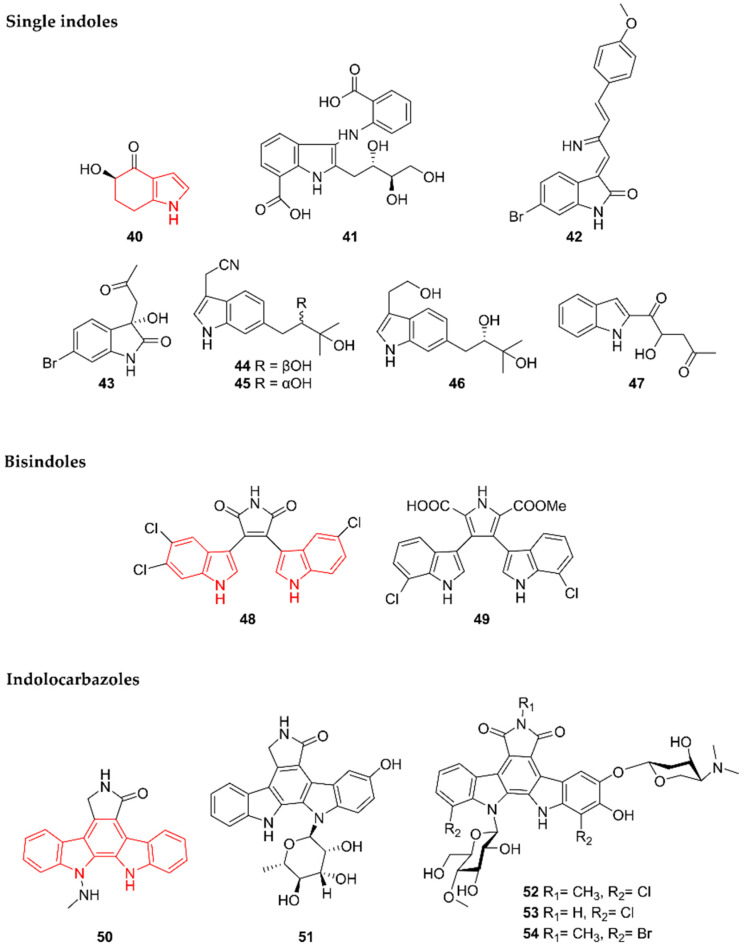
The single indole alkaloids microindolinone A (**40**), anthranoside C (**41**), saccharomonosporine (**42**), convolutamydine F (**43**), streptoprenylindoles A–C (**44**–**46**), and 11457B (**47**). The bisindole alkaloids dionemycin (**48**) and 6-OMe-7′,7′′-dichlorochromopyrrolic acid (**49**). The indolocarbazole alkaloids 12-N-methyl-k252c (**50**), 3-hydroxy-K252d (**51**), and loonamycins A–C (**52**–**54**). The structural alkaloid groups are represented in red.

Indoles are among the best-studied alkaloids in the marine environment, which is reflected by the number of discovered compounds of this class. While this review focuses on alkaloids with an Actinobacteria origin, alkaloids produced by marine fungi after 2015 have been covered by Willems et al. (2020) [11]. An exhaustive review on marine indoles unraveled prior to 2015 is also available [38]. In this paper, indoles are subdivided into three subgroups: single indoles, bisindoles, and indolocarbazoles (Figure 9).

#### 2.9.1. Single Indoles

Microindolinone A (**40**) was discovered by Niu et al. (2017) in extracts from *Microbacterium* sp. MCCC 1A11207, a strain isolated in 2014 from a sample of deep-sea sediment from the south-western Indian Ocean taken at a depth of 1.6 km [39]. An antiproliferative and anti-allergic activity test showed that **40** is not cytotoxic for RBL-2H3 cells and does not alleviate allergic reactions. The β-hexosaminidase activity was quantified in RBL-2H3 cells after treatment with 20 µg/mL of **40**. The authors did not report antimicrobial or other cytotoxic effects of **40**.

In 2018, Che et al. identified anthranoside C (**41**) from *Streptomyces* sp. CMN-62 [40]. This strain was collected from an unidentified sponge sample from Naozhou Island in the Guangdong Province in China. Compound **41** showed activity against influenza A H_1_N_1_, with an IC_50_ of 68.46 µg/mL. Additionally, cytotoxicity and NF-κB inhibitory tests were performed but no activity was detected. Furthermore, a biosynthesis pathway using anthranilic acid (ATA) and D-glucose (one of the major constituents of the culture medium) as building blocks was proposed. Anthranoside A and B, compounds also identified from the same strain, were proposed to be the result of a concession of D-glucose and ATA, followed by an Amadori rearrangement and ketalization. Subsequent condensation and cyclization were reported as leading to the formation of **41**.

Saccharomonosporine A (**42**) and convolutamydine F (**43**) were discovered by El-Hawary et al. (2018) [41]. These indole alkaloids were obtained by co-culturing the two actinomycetes *Saccharomonospora* sp. UR22 and *Dietzia* sp. UR66, isolated in 2015 from the Red Sea sponge *Callyspongia siphonella* in Egypt at a depth of 10 m. Compounds **42** and **43** were not detected in the strains when grown separately, indicating co-culturing is crucial for expression. Both compounds were brominated, which is unique compared to the other (new) alkaloids from marine actinomycetes. Only **42** showed potent antiproliferative activities towards HL-60 and HT-29 cells. The authors suggested that the cytotoxicity is obtained through the inhibition of the oncoprotein Pim-1 kinase, because **42** significantly reduced this enzyme’s activity.

Streptoprenylindoles A–C (**44**–**46**) were discovered by Yi et al. (2019) in extracts from *Streptomyces* sp. ZZ820 [42]. This strain was retrieved from a sea coastal soil sample collected in the East China Sea. The compounds were solely screened for antimicrobial activity against MRSA and *E. coli*, but were unsuccessful.

Together with the pyrimidine 11457A (**37**), Fang et al. (2019) reported the indole 11457B (**47**) from *Pseudonocardia* sp. SCSIO 11457 [35]. Compound **47** was subjected to the same screening tests as 11457B but did not demonstrate any activity.

#### 2.9.2. Bisindoles

In addition to single indoles, dimerization of these moieties resulting in bisindoles was observed as well. Song et al. (2020) unraveled two bisindoles, dionemycin (**48**) and 6-OMe-7′,7′′-dichlorochromopyrrolic acid (**49**), derived from *Streptomyces* sp. SCSIO 11791, isolated from a sediment sample taken at a depth of 1.7 km in the South China Sea [43]. Both compounds had a moderate cytotoxic effect on MDA-MB-435 human breast cancer cells, with IC_50_ values of 1.68 and 9.08 µg/mL, respectively. In addition, MDA-MB-231 human breast adenocarcinoma cells, HCT-116 colon cancer cells, NCI-H460 human non-small-cell lung cancer cells, HepG2 liver cancer cells, and non-cancerous MCF10A human breast epithelial cells were screened and yielded IC_50_ values in the range of 1.36 to 11.7 µg/mL. In comparison, **48** exhibited superior activity. A possible explanation for the difference in efficacy could be due to chlorination at different positions. Finally, the compounds’ antibacterial activities were evaluated against Gram-positive bacteria (*Micrococcus luteus*, *S. aureus*, and MRSA cells isolated from pigs and humans) and Gram-negative bacteria (*A. baumannii*, *Vibrio coralliilyticus*, and *V. alginolyticus*). Both **48** and **49** showed inhibitory effects against the Gram-positive bacteria, but not against the Gram-negative bacteria [43]. Again, the MIC values of compound **48** were more promising than those of **49**, indicating that the position of halogenation has a possible effect on cytotoxicity as well as on antimicrobial activities.

#### 2.9.3. Indolocarbazoles

Indolocarbazoles are a subgroup of bisindoles bearing an indolo(2,3-a)pyrrole(3,4-c)carbazoles structure, often associated with a sugar residue [44]. They are known as potent protein kinase and topoisomerase I inhibitors [45] and hence gained a lot of interest for cancer treatment. The compound 12-N-methyl-k252c (**50**) was identified by Cheng et al. (2018) [46] as a product of *Streptomyces* sp. A22, which was found in marine sediments of Ningbo County in China. BRD4 inhibitory activities, protein kinase inhibition activities, and cytotoxic activities against PC3 human cancer cells were evaluated. However, compound **50** only showed activity against protein kinases with IC_50_ values ranging from 0.31 to 0.63 µg/mL.

Using a precursor-directed approach, Wang et al. (2018) succeeded in obtaining the new indolocarbazole 3-hydroxy-K252d (**51**) [47]. This compound was isolated by adding 5-hydroxy-L-tryptophan to a culture of *Streptomyces* sp. OUCMDZ-3118, a strain isolated from a deep-sea sediment taken at depth of 2 km in the South China Sea. Cytotoxic activities tested against A549, MCF-7, and K562 tumor cells showed moderate activity.

In 2020, the indolocarbazoles loonamycins A–C (**52**–**54**) were discovered by means of genome mining [48]. All three compounds were produced from *Nocardiopsis flavescens* NA01583, recovered from marine sediment in Yongxing Island, China. Next to possessing halogen atoms, the compounds comprise sugar moieties. Based on the information obtained from the bioinformatic analysis, a proposal was made for their biosynthesis pathway. Starting from tryptophan, four core enzymes, namely the chlorinating LooO, the dimerizing LooD, the condensating LooP, and the decarboxylating LooC, together with tailoring enzymes such as a glycosyltransferase, contribute to the formation of **52**–**54**. Only **52** displayed cytotoxic activities against eight cell lines including SH-SY5Y (neuroblastoma), Sum1315 (breast cancer), HT29 (colorectal cancer), HCT116 (colorectal cancer), Hela (cervical cancer), SW872 (liposarcoma), and HCC78 (lung cancer). IC_50_ values ranging from 29.65 ng/mL to 204.69 ng/mL were obtained, indicating that **52** is very promising.

### 2.10. Diketopiperazines

**Figure 10:**
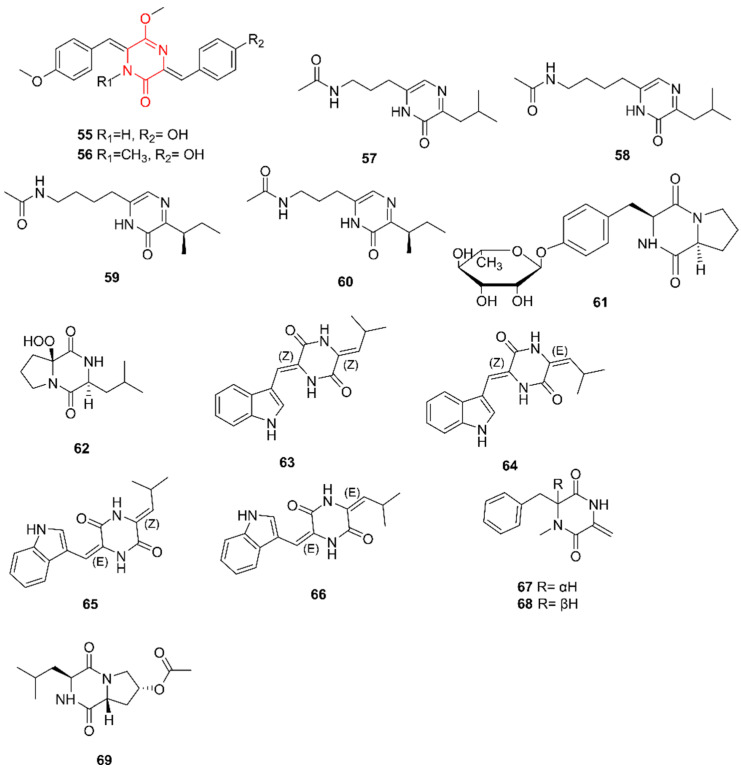
The diketopiperazine alkaloids nocazines F and G (**55**–**56**), streptopyrazinones A–D (**57**–**60**), maculosin-O-α-L-rhamnopyranoside (**61**), actinozine A (**62**), photopiperazines A–D (**63**–**66**), streptodiketopiperazines A and B (**67**–**68**), and cyclo-(D-8-acetoxyl-Pro-L-Leu) (**69**). The structural alkaloid group is represented in red.

In total, 14 new diketopiperazines (DKPs) have been identified from marine actinomycetes since 2017 (Figure 10). DKPs are cyclic dipeptides, resulting from the condensation of two α-amino acids, which have piqued interest due to their broad range of activities. In addition to their use as antimicrobials, antivirals, and antitumor agents, DKPs can be used in the treatment of ischemic brain injury [49], can interfere with quorum-sensing signaling [50], and are promising candidates for the treatment of neurodegeneration-related pathologies, such as Alzheimer’s disease [51]. Furthermore, they possess a stable heterocyclic scaffold with restricted conformation, meaning that DKPs control their stereochemistry at up to four positions [52].

Nocazines F and G (**55**–**56**) were discovered by Sun et al. (2017) in extracts from *Nocardiopsis* sp. YIM M13066, collected from a deep-sea sediment (location unknown) [53]. Both were screened for cytotoxic activities against the human cancer cell lines H1299, HeLa, HL7702, MCF-7, PC3, and U251 and for antibacterial activities against *S. aureus*, *B. subtilis*, *P. aeruginosa*, and *Salmonella enterica* serovar *typhimurium*. They were both cytotoxic for all tested cell lines, but only **56** was moderately active against *B. subtilis*. Additionally, **55** seemed to exert a strong inhibitory effect on the type-three secretion system (T3SS) of *S. enterica* serovar *typhimurium*.

Chen et al. (2018) discovered four new DKP derivatives, streptopyrazinones A–D (**57**–**60**), produced by *Streptomyces* sp. ZZ446, a strain isolated from the coastal soil of the Zhoushan Islands in China [54]. To overcome the problem of silent gene clusters, ten different media were used. All four compounds were screened for activity against MRSA, *E. coli*, and *C. albicans* and for cytotoxic effects on glioma U87MG, U251, SHG44, and C6 cells. However, only activity against MRSA and *C. albicans* was detected. A more thorough investigation of the same strain led to the discovery of maculosin-O-α-L-rhamnopyranoside (**61**), which was the first glycosylated DKP collected from a natural source [55]. This compound was screened for antimicrobial activity against MRSA, *E. coli*, and *C. albicans*, and was active against all three. Comparing these results with those for streptopyrazinones A-D indicates that the glycosylation of a DKP could influence efficacy.

In addition to the two new nucleoside analogues described above, Shaala et al. (2019) discovered actinozine A (**62**) in extracts from the *Streptomyces* sp. strain Call-36 [37]. This compound was subjected to the same cytotoxicity screening and also demonstrated weak activity against breast cancer cell lines. Compound **62** was also screened for antimicrobial activities against *S. aureus* and *C. albicans* and was active against both.

Photopiperazines A–D (**63**–**66**) were isolated by Kim et al. (2019) from *Streptomyces* AJS-327, collected from an unidentified sponge fragment in Scripps Pier in La Jolla, California [56]. As the four isomer compounds interconvert after light exposition, a mixture of the four was used to evaluate cytotoxic properties, which showed potent and selective growth inhibition of SKOV3 ovarian cancer cells and human U87 glioblastoma brain cancer cells.

In 2020, streptodiketopiperazines A and B (**67**–**68**) were discovered by Yi et al. in extracts from *Streptomyces* sp. SY1965, isolated in 2018 from the Mariana Trench at a depth of 11 km [57]. This actinomycete lives in a unique habitat with a high pressure, in contrast to the other isolated actinomycetes. Compounds **67** and **68** both showed antifungal activity against *C. albicans*, with a MIC of 42 µg/mL. Both compounds did not show antiproliferative activity against human glioma U87MG and U251 cells, and no antimicrobial activities against MRSA or *E. coli* were detected either.

Finally, Song et al. (2020) elucidated the structure of cyclo-(D-8-acetoxyl-Pro-L-Leu) (**69**), isolated from *Streptomyces* sp. SCSIO 41400 [58]. This strain was obtained from mangrove soil from the Fuli Mangrove Bay Wetland Park, Haikou, Hainan Province of China. Compound **69** was evaluated for inhibitory activity against AChE and pancreatic lipase and yielded an IC_50_ value of 27.3 µg/mL for the latter.

### 2.11. Quinolines

**Figure 11:**
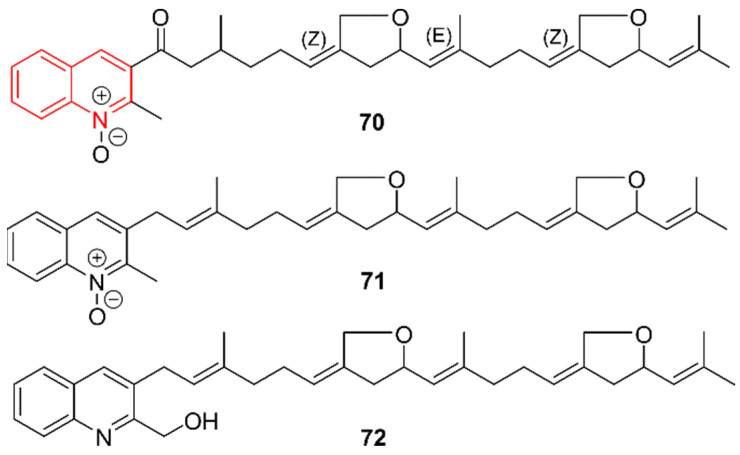
The quinoline alkaloids marinoterpins A–C (**70**–**72**). The structural alkaloid group is represented in red.

In total, three new quinolines were discovered in marine actinomycetes (Figure 11). Kim et al. (2021) identified the linear merosesterterpenoids marinoterpins A–C (**70**–**72**), which originated from two different strains [59]. Compound **70** was isolated from an enhanced investigation of *Streptomyces* AJS-327, a strain that also led to the discovery of a DKP in 2019, as described earlier. Compounds **71** and **72** were derived from *Streptomyces* CNQ-253, isolated from a marine sediment sample collected at a depth of 46 m in San Diego, California. Instead of testing for antimicrobial activities or cytotoxic effects, the elucidation of the marinoterpin biosynthetic gene cluster was the main objective. Based on the biosynthetic knowledge of aurachins A-D, compounds that show similarity to **70**–**72**, a proposal was made. The quinoline core was synthesized by a sesterterpene diphosphate synthase, a prenyltransferase, two β-ketoacyl-acyl carrier protein (ACP) synthases, an ACP, and a benzoate-CoA ligase. Additionally, a cytochrome P450, a flavin monooxygenase, an FAD-dependent oxidoreductase, an NAD-dependent epimerase, and a methyl-transferase core enzyme were proposed for subsequent derivatization.

### 2.12. Isoquinolines

**Figure 12:**
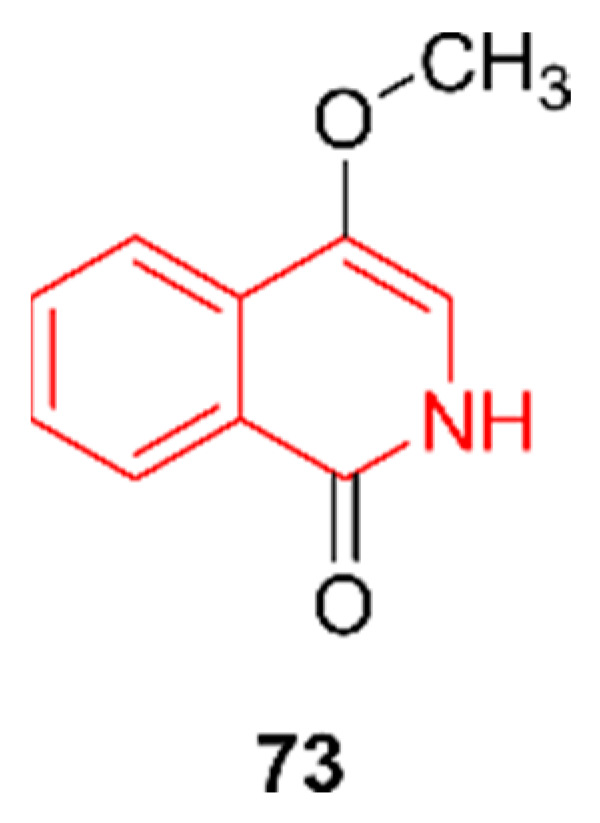
The isoquinoline alkaloid isoquinoline 4-methoxy-2H-isoquinolin-1-one (**73**). The structural alkaloid group is represented in red.

Isoquinoline derivatives are known to have anticancer effects as they are capable of binding to the estrogen receptor alpha and VEGF receptor 2, both of which have an important role in breast cancer [60]. The isoquinoline 4-methoxy-2H-isoquinolin-1-one (**73**, Figure 12) was discovered by Eliwa et al. (2017) in extracts from *Nocardiopsis lucentensis* sp. ASMR2, a strain originating from a marine plant in the Red Sea in Egypt [61]. Antimicrobial activity was tested against *P*. *aeruginosa*, *B. subtilis*, *S. aureus*, *C. albicans*, *Saccharomyces cerevisiae*, and *Aspergillus niger*; however, no activity was detected. Cytotoxicity against a human cervix carcinoma cell line (KB-3-1) and its multidrug-resistant subclone (KB-V1) was evaluated, but the tests yielded no activity.

### 2.13. Hybrid Alkaloids

**Figure 13:**
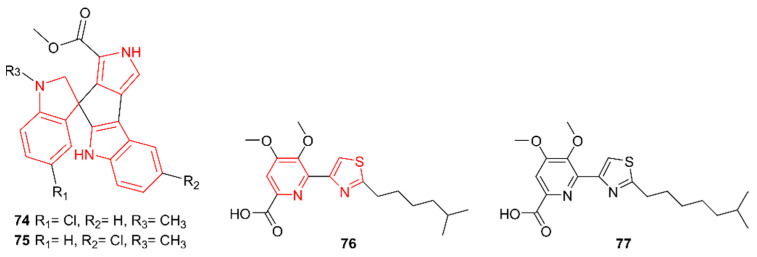
The hybrid alkaloids spiroindimicins E and F (**74**–**75**) and pyrizomicin A and B (**76**–**77**). The structural alkaloid groups are represented in red.

In recent years, several alkaloid hybrids composed of more than one alkaloid core structure have been unraveled (Figure 13). Paulus et al. (2017) discovered spiroindimicins E and F (**74**–**75**) from *Streptomyces* sp. MP131-18, a strain isolated from a marine sediment sample collected in the Trondheim Fjord, Norway [62]. These compounds have both a pyrrole and a bisindole core. They were discovered through genomics-metabolomics profiling, whereby the biosynthetic gene cluster information was mapped to the secondary metabolites profile of the strain. The biosynthesis involved a tryptophan 2-monooxygenase, a chromopyrrolic acid synthase, and a cytochrome P450, three core enzymes necessary for the formation of bisindole pyrroles. Only **74** was tested for antimicrobial activity against *B. subtilis*, *E. coli*, and *Pseudomonas putida*, yet no activity was detected. Within the group of spiroindimicins, the degree of halogenation seemed to influence antimicrobial activity. For example, spiroindimicin B contains two halogens and possesses weak antimicrobial activity. In contrast, compound **74** has only one chlorine moiety, possibly explaining its lack of antimicrobial activity. In addition, **74** showed minor cytotoxic effects on T24 bladder carcinoma cells.

Analysis of the rare actinomycetes strain *Lechevalieria aerocolonigenes* K10-0216, isolated in 2014 from a mangrove sediment sample from Iriomote Island in Japan, led to the discovery of pyrizomicin A and B (**76**–**77**), which contain a thiazole as well as a pyridine skeleton [63]. These compounds both exhibit antimicrobial activity against *B. subtilis*, *Kocuria rhizophila*, *E. coli*, *Xanthomonas campestris* pv. Oryzae, and *C. albicans* but are not active against *Mucor racemosus* IFO4581.

## 3. Alkaloid Discovery Strategies

### 3.1. Wet-Lab Strategies

While Actinobacteria have been the subject of many functional screens or extraction analyses, plenty of interesting secondary metabolites are still found when studying them. However, the discovery of novel compounds over known metabolites is not straightforward. The following section contains a thorough review of the wet-lab workflows used to find the new alkaloids discussed in this review. A detailed overview of the applied strategies is provided in Supplementary Table S2.

#### 3.1.1. Culturing Strategies

The circumstances in which the natural production host is grown are key in the hunt for novel compounds through wet-lab strategies. Changing the strain/isolate, the growth medium or other culturing conditions can have a huge effect on success.

Different microbial isolates can be screened prior to in-depth strain characterization to maximize the likelihood of discovering novel secondary metabolites, while decreasing the risk for compound rediscovery. Strain prioritization by metabolomics (SPM), in some articles also referred to as ‘physicochemical screening’ (PC), is a promising strategy to address this hurdle as it allows for comparative metabolomics, revealing strains with unique metabolites [64,65]. For example, the analysis of metabolic fingerprints of 72 *Micromonospora* sp. extracts resulted in the detection of phallusialides A-E [29]. A comparison of metabolic profiles also led to the discovery of the novel alkaloids pyrizomicin A and B [63].

From the articles discussed here, no general strategy pertaining to desired medium components can be distilled. Still, complex media and sea salt or sea water are often applied, while a neutral pH (7.0–7.4) is preferred at times. As marine Actinobacteria thrive in a halogenated environment, their secondary metabolites often possess halogen atoms. The use of different salts in the medium is therefore a viable strategy to discover new alkaloids. In the case of loonamycins A–C, NaBr was supplemented to the medium to force the incorporation of bromide atoms into the secondary metabolites [48]. Similarly, a shift in the ratio of KBr:NaCl added to the growth medium yielded the brominated phallusialide B [29]. A more direct method is feeding a precursor analogue to the culture medium, which has led, for example, to the discovery of 3-hydroxy-K252d by adding 5-hydroxy-L-tryptophan [47]. Interestingly, solid particles such as glass beads, Diaoin HP-20 (polystyrene), sterilized rice, or calcium carbonate were added to the fermentation medium on occasion. Finally, using different culture media can activate new biosynthetic gene clusters (BGCs), as applied in the ‘one strain, many compounds’ (OSMAC) strategy, which has led, for example, to the discovery of the four new marine alkaloids streptopyrazinones A−D [54].

Apart from changing the medium or adding a trigger compound, dormant gene clusters can also be activated through co-culturing [66,67]. In the case of a microbial co-culture of the marine actinomycetes *Saccharomonospora* sp. UR22 and *Dietzia* sp. UR66, two new alkaloids were observed: saccharomonosporine A and convolutamydine F [41]. Such co-culturing leads to a competition for nutrients as well as crosstalk between both species, causing altered microbial metabolisms compared with axenic cultivation.

#### 3.1.2. Extraction and Isolation Strategies

Mostly, the extraction of (new) alkaloids from microbial culture broth relies on the affinity of these secondary metabolites for a solvent over aqueous phase, i.e., liquid–liquid extraction (LLE). The solvents used are often methanol, ethanol, acetone, ethyl acetate, butanone, (cyclo)hexane, or a combination of these. The extraction step is preceded by a filtration step to separate microbial cells from the broth. While liquid cultivation is the principal method, in the case of maculosin-*O*-α-L-rhamnopyranoside, a solid medium was used, entailing a cutting of the solid culture instead of filtration prior to extraction [55]. The compounds phallusialides A − E were extracted using solid-phase extraction (SPE). The polystyrene particles (Diaion-HP20) were filtered from the broth and extracted with acetone [29]. Though extraction of the extracellular broth is often preferred, extraction of the mycelia with the same solvents was performed for several alkaloids to obtain a higher yield or to retrieve intracellular compounds. The extraction steps are repeated frequently, and samples can be concentrated through subsequent rotary evaporation. As the culturing of Actinobacteria generally makes use of complex media components, LLE and SPE also remove constituents with an aqueous affinity to improve fractionation of the extracts.

Indeed, following extraction of fermentation broth or mycelia, samples can be fractionated to limit the number of metabolites in a single analysis, enabling separation and, thus, the detection of novel compounds. Such fractionation is typically undertaken through repeated column chromatography, albeit in combination with, for example, preparative thin layer chromatography (TLC), though the latter is becoming more and more outdated for use in these circumstances. This multi-dimensional chromatographic separation is based on SPE using multiple columns with different stationary phases, e.g., a silica gel, a dextran-based resin (i.e., Sephadex LH-20), or C18 packing column (ODS). Mobile phases often consist of methanol, acetonitrile, chloroform, ethyl acetate, cyclohexane, or petroleum ether used with a gradient or isocratic elution. In addition, supercritical fluid chromatography and ion exchange chromatography have been described in literature as being used to separate alkaloids [68]. The streptoprenylindoles A and B are enantiomers and required chiral derivatization with the Mosher’s reagent prior to the final column chromatography step [42]. Specifications of compound isolation strategies used are given in Supplementary Table S2.

Finally, further purification was generally conducted prior to NMR analysis using a (semi)-preparative HPLC using a C18 column and as mobile phase MeOH or acetonitrile combined with H_2_O.

#### 3.1.3. Compound Identification

Chemical structure elucidation is essential to determine the uniqueness of novel alkaloids. As one general analytical technique is insufficient to unequivocally assign a chemical structure, a combination of different technologies is a prerequisite. The novel marine alkaloids discussed in this manuscript were subjected to ^1^H, ^13^C, and 2D-NMR analyses (homonuclear correlation spectroscopy (COSY), heteronuclear multiple bond correlation (HMBC), heteronuclear multiple-quantum correlation (HMQC), heteronuclear single quantum correlation (HSQC), distortionless enhancement by polarization transfer (DEPT), nuclear overhauser effect spectroscopy (NOESY), and rotating-frame overhauuser effect spectroscopy (ROESY) combined with high-resolution mass spectrometry (HRMS) to elucidate their structure. Additionally, infrared (IR), ultraviolet (UV), and electronic circular dichroism (ECD) spectral data were sometimes acquired. Tandem mass spectrometry (MS/MS) was employed to identify streptopertusacin A, actinozine A, thymidine-3-mercaptocarbamic acid, and thymidine-3-thioamine and phallusialides A−E [22,29,37]. In the case of streptodiketopiperazines A and B, chartrenoline, streptoglutarimides A−J, anthranosides A−C, streptopyrazinones A−D, anandins A and B, and strepchazolin A–B, X-ray diffraction analysis was conducted as well [15,17,20,30,40,54,57]. While most studies used a combination of analytical techniques and were able to retrieve sufficient methodical information to reproduce their results, the elucidation of some novel compounds is questionable. For example, dionemycin and 6-OMe-7′,7″-dichorochromopyrrolic acid were analyzed solely using a 2D-NMR analysis (HMBC) [43]. IR, UV, and ECD spectra were not conducted to verify the structures for the identification of the novel nocazines [53]. Finally, no UV or IR spectra were measured to clarify 2-ethylhexyl 1H-imidazole-4-carboxylate, 4-methoxy-2H-isoquinolin-1-one, microindolinone A, or 11457 A-B, which implies less reliable results [33,35,39,61]. In addition, photopiperazines C and D were characterized by 2D-NMR analysis without prior purification, because of the small amounts present in the extracts [56]. A detailed overview of the employed alkaloid identification techniques is given in Supplementary Table S2. Careful attention should thus be given to the quality of its proposed structure.

### 3.2. Genome Mining

Though successfully applied, finding novel alkaloids through wet-lab strategies remains a challenging task. For example, known metabolites often obscure screening strategies. This is evermore the case with actinomycetes, as about 70% of the known microbial secondary metabolites are produced by this microbial family, with *Streptomyces* sp. being their main source [69]. Another problem resides in the fact that the responsible BGCs can be silent under laboratory conditions [70]. To overcome this conundrum, approaches such as microbial co-culturing [41] or the isolation chip (iChip) [71] have been successfully applied. Nevertheless, circumventing the stringent regulation of BGCs can require a lot of effort, as the triggers for production are not always easily found. Alternatively, genome mining is gaining traction due to the continuous technological progress of reliable genome sequencing and assembly, due to the better understanding of the biochemical origin and BGCs of natural products and the plethora of computational tools available for genome annotation and gene cluster identification [72].

The quality of genome sequences and assemblies is of paramount importance to enable reliable genome mining [73]. The next generation sequencing (NGS) platforms of Illumina, considered the gold standard of short-read sequencing methods (100–300 bp), have a low error rate (1–2% errors) [74] but are of limited efficiency to assemble large or complex genomes [75]. In comparison, long-read sequencing methods such as those of PacBio and Oxford Nanopore Technologies return much longer reads, which facilitates genome assembly. In the early days, these technologies had a higher error rate, warranting a sufficient coverage, but this has recently been overcome. Long read, high quality genomic sequences untangle difficulties during assembly, especially for large BGCs containing genes with repetitive domains [76]. In hybrid technologies, such long-read sequences, information can provide a scaffold for the accurate short reads resulting from NGS, in the end leading to a higher quality of genome [77]. Low-quality genome sequences where genomes are instead assembled into contigs can impede accurate coding sequence prediction through frameshifts, significantly impacting the outcome of genome mining. Indeed, mistakes during assembly frequently result in annotation errors; they are the cause of a diverging cascade of faulty annotations in (public) genome databases [78].

In practice, assembly on long-read sequence data can be performed using tools such as Flye, Canu, Miniasm/Minipolish, Raven, Redbean, and Shasta [79], whereas short-read assembly can be completed using tools such as ABySS [80] and MaSuRCA [81]. A more in-depth comparison of long-read assembly tools is given by Wick et al. (2019) [79].

Apart from correctly constructing an assembled genome, an additional hurdle to overcome is the correct annotation of coding sequences, even with high quality genomes. Prokka [82] and PGAP [83] are popular tools used for annotation. For a deeper understanding of different annotation tools used for microorganisms, the reader is referred to the review of Dong et al. (2021) [84]. Assembly and annotation are crucial steps that have an impact on the outcome of genome mining, and therefore, these steps should be performed thoughtfully.

The set of computational toolboxes used to unravel (new) secondary metabolite BGCs, making predictions based on highly conserved regions, is continuously evolving. Established tools such as PRISM and antiSMASH make use of a sequence alignment-based profile in a Hidden Markov Model (HMM) of genes that is characteristic for certain BGCs [85,86]. Specifically, for actinomycetes, antiSMASH is the most widely used tool for identifying and analyzing secondary metabolite BGCs. BAGEL, ClustScan, and CLUSEAN are other available tools, but they are limited to only certain classes of secondary metabolites, making them less popular [87]. With the advent of machine learning for biotechnological purposes, tools such as ClusterFinder and DeepBGC were designed to increase the probability of identifying as yet unknown BGCs instead of focusing on specific genes. ClusterFinder first compresses annotated genome sequences to a string of protein family (Pfam) domains, followed by clustering the genes based on the Pfam domain information [88]. In contrast to antiSMASH, ClusterFinder can be used without a priori knowledge of signature enzymes. This method still relies on HMMs, meaning that position dependency effects between distant genomic entities are ignored. The use of vector versions of Pfam domains and a deep learning approach using Recurrent Neural Networks, allows DeepBGC to remember these position effects, resulting in a peaked level of novel BGC classes and improved detection accuracy [89]. A more in-depth comparison of computational tools used during genome mining is given in the review of Chavali et al. (2018) [90].

Genome mining strategies can generally be divided in two approaches [87]. In the forward approach, the BGC is unraveled from the genome with computational tools, followed by an attempt to predict the chemical structure of the resulting metabolite. This approach is currently gaining interest as the available genomic information is vastly expanding. However, there are two main bottlenecks. On the one hand, the draft structure of the metabolites needs to be predictable to allow structural confirmation through, e.g., mass spectrometry or NMR, validating the functionality of the hypothetical BGC. Training databases linking biosynthetic enzymes to chemical transformations exist but do not yet reflect the entire biological diversity of natural product biosynthesis, causing limitations in structure prediction [91]. On the other hand, stringent regulation and the low expression of BGCs of interest can lead to low metabolite concentrations, hampering detection and structure confirmation. The heterologous expression of the BGC of interest in a carefully selected non-native strain yields the potential to address the latter hurdle by circumventing regulation or through a reduced metabolic background. In the reverse approach, the gene cluster for a known metabolite is sought. In contrast to the forward approach, the likelihood of discovering silent BGCs is lower in the reverse approach, reducing its power in this regard. Paradoxically, these forward and reverse approaches connect with each other. Indeed, the reverse method is an indispensable step, as it feeds the BGC databases, which is beneficial for the forward approach. Many novel compounds described in this review were not analyzed for their corresponding BGC, missing opportunities to further enhance smBGC insights. Recent advances have combined mass spectrometry data (reverse approach) with genomic information (forward approach) to unravel multiple compound-BGC linkages at the same time. This technique is discussed in more depth in the review of Crüsemann (2021) [92].

**Figure 14:**
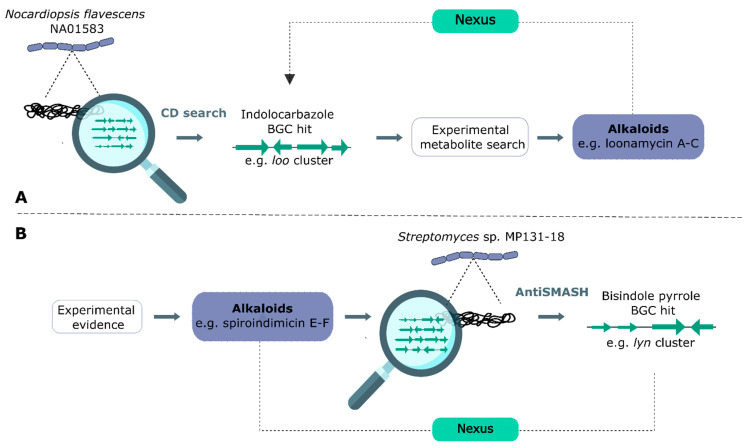
In general, genome mining can be conducted in two different ways. (**A**) Starting from the genome sequencing data of a strain of interest, a conserved domain (CD) search leads to BGC hits. Subsequently, an extensive experimental metabolite search leads to the compound associated with the BGC hit, e.g., loonamycin A–C in the case of Yang et al. (2020) [48]. Having this information, the nexus can be determined between the metabolite and BGC by, for example, pathway prediction and linking biosynthetic enzymes to chemical transformations. (**B**) Starting from novel metabolites, genomic analysis can lead to the BGC of the corresponding metabolite. Finally, the nexus between the BGC and metabolite can be analyzed. This method was used by Paulus et al. (2017), leading to the identification of spiroindimicin E–F and its BGC [62].

After sequencing the genome of *Nocardiopsis flavescens* NA01583, Yang et al. (2020) were able to elucidate the *loo* BGC by making use of the known architecture of indolocarbazole alkaloid gene clusters [48]. These clusters generally contain four signature enzymes (RebO/StaO, RebD/StaD, P450 RebP/StaP, and RebC/StaC) necessary to create the core structure of indolocarbazoles. Using these signatures, the *loo* cluster was discovered. Subsequently, an extensive experimental metabolite search led to the isolation of three novel alkaloids**,** loonamycin A–C. Furthermore, blasting of the cluster led to the identification of tailoring enzymes, which led to the proposal of the hypothetical biosynthetic pathway. Structural derivatization occurs through tailoring enzymes such as halogenases, methyltransferases, and glycosyltransferases, but these activities are less suitable for genome mining purposes. Validation of the pathway was achieved by implementing the *loo* cluster in the heterologous *Streptomyces lividans* K4-114 host, yielding loonamycin C. Gene knockouts of the *loo* cluster also confirmed its involvement in the production of these indolocarbazoles. A graphical representation of this genome mining approach in general can be found in Figure 14A. Contrary to this BGC-based genome mining approach, the bisindole pyrrole *lyn* cluster of spiroindimicin E–F was elucidated after the chemical structure of these compounds was determined [62] (Figure 14B). To do so, the authors used Illumina sequencing to unravel the genome of *Streptomyces* sp. MP131-18, in which they identified 36 BGCs using antiSMASH. Linkage of the chemical structure of these bisindole–pyrrole metabolites with the *lyn* cluster was also based on known signatory enzymes. Although validation of this BGC was not performed, deletion of the cluster of interest or its heterologous expression is pivotal to prevent a cascade of annotation errors and hence genome mining errors.

While cluster deletion or heterologous expression by a natively non-producing host are generally employed for BGC confirmation, several other, less conclusive, approaches exist. For example, instead of deleting one or more of the genes in the cluster, gene mutants can be introduced to block the synthesis of the product of interest [93]. Moreover, precursor feeding strategies based on the hypothetical biosynthesis pathway provide evidence of the validity of the genome mining predictions. This way, partial evidence on alkaloid biochemistry can be provided, potentially leading to the elucidation of novel compounds, as was the case with argimycins P.

Though progress is being made on genome mining for novel alkaloids, often relying on the identification of NRPS and PKS clusters, biosynthesis pathways are frequently puzzling or obscured, resulting in few hypothetical biochemical synthesis routes being proposed by authors.

### 3.3. Synthetic Biology in Alkaloid Discovery

Over the past decades, the maturation of synthetic biology did not only elevate the development of microbial cell factories but also influenced natural product discovery strategies.

While most actinomycetes contain over 20 BGCs per genome, their functional expression under laboratory conditions remains a major bottleneck [94]. Synthetic biology is capable of activating silent BGCs for discovery in several ways [95]. First, the transfer of the BGC from the native producer to a (closely related) heterologous host potentially alleviates the strict regulation present in the natural producer. For actinomycetes, multiple heterologous hosts with reduced secondary metabolism or with engineering advantages have been developed. Examples include *Streptomyces lividans*, *S. coelicolor*, *S. avermitilis*, *S. venezuelae*, and *S. albus* [96,97]. For example, the *loo* cluster was heterologously expressed in *S. lividans* for loonamycin C production [48]. Second, multiplexed refactoring can be applied to express silent BGCs, optimizing the transcription of BGCs through the simultaneous replacement of multiple native promoters with synthetic counterparts [98]. Next, the transcription of silent BGCs in *Streptomyces* sp. can be triggered by altering the expression of regulatory genes. Strict transcriptional regulation can be circumvented by introducing a global regulator which activates silent BGCs or eliminating downregulating genes [99]. In this regard, the alkaloids geranylpyrrol A and piericidin F have been produced by *Streptomyces* sp. CHQ-64 ΔrdmF [27]. Finally, ribosome engineering has proven to be valuable in upregulating silent clusters. An in-depth overview of these transcription-based strategies to activate silent BGCs is given by Liu et al. [99].

In addition to fiddling with transcriptional regulation, synthetic biology provides multiple options on a translational level as well. Due to their high GC-content (>70%), Actinobacteria have a different codon usage profile than many other microorganisms. Upon the introduction of BGCs into a heterologous host, translational issues often occur, because of codon usage bias. AT-rich codons are, for example, rare in Actinobacteria, while they might be more common in the heterologous host. Synthetic biology thus allows the rational design of the coding sequence of BGC genes through the selection of the most fitting synonymous codon for the heterologous host [100]. Such codon optimization or harmonization approaches have gained increasing interest over the years, as they often lead to an improved translation rate, yielding more correctly folded proteins and hence potentially more metabolite. In addition, the ribosome binding site (RBS) present in promoter sequences contains the Shine–Dalgarno (SD) sequence, which is short and crucial in translation efficiency, making it an ideal target for developing synthetic RBSs and, hence, playing with translation levels [95].

In contrast to RBSs, riboswitches regulate both transcription and translation processes through ligand-binding, making these ideal antimicrobial targets. Subsequently, new antimicrobial drugs can be discovered through the screening of synthetic ligands using fragment screening, high-throughput screening, and rational design of structural analogues [101]. To this end, synthetic biology approaches can be of great help.

## 4. Conclusions

The marine environment has long been underrepresented in natural product discovery. However, between 2017 and mid-2021, 77 novel alkaloids from marine Actinobacteria were reported, of which the indoles (15), diketopiperazines (15), glutarimides (10), indolizidines (10), and pyrroles (8) represent the majority. In vitro assays with these alkaloids, e.g., testing their antimicrobial activity or cytotoxicity, confirm the significance of secondary metabolites produced by marine actinomycetes. Although the marine environment is gaining increased attention, a major geographical bias is in place as most discoveries occurred in China.

The alkaloids discussed in this review were mainly discovered by fractioning extracts of marine actinomycetes to isolate compounds prior to chemical structure elucidation. A combination of multiple experimental strategies, such as precursor feeding and metabolomics, has led to the unearthing of new alkaloids. However, the extraction and isolation methods used are outdated. Therefore, additional attention should be given to novel and greener strategies. Likewise, the structure determination of several alkaloids is unreliable and requires more thorough investigation, using methods which make more frequent use of the state-of-the-art technologies available.

While experimental discoveries make up the bulk of discoveries, genome-mining approaches are gaining interest due to the maturation of sequencing technologies and mining tools. Annotation remains the main hurdle, as most genome sequences in public databases are of lesser quality, resulting in a cascade of erroneous annotations.

Finally, synthetic biology holds the potential to aid drug discovery strategies as well. BGCs unraveled through genome mining can be refactored and implemented in heterologous hosts to circumvent strict regulation in the natural producer. Moreover, the removal or overexpression of regulatory genes has proven useful. At a translational level, synthetic biology has the power to counter codon bias issues. 

## Supplementary Materials

The following are available online at https://www.mdpi.com/article/10.3390/md20010006/s1: Table S1: Newly discovered alkaloids from marine Actinobacteria between 2017 and mid-2021, Table S2: Employed experimental strategies to discover new marine alkaloids from Actinobacteria.
